# Masson's Hemangioma Mimicking As Leaking Aortic Pseudoaneurysm: An Extremely Rare Presentation

**DOI:** 10.1055/s-0039-1688925

**Published:** 2019-09-17

**Authors:** Mayank Yadav, Khushwant Popli, Akshay Kumar Bisoi, Sandeep Chouhan

**Affiliations:** 1 Department of Cardiothoracic and Vascular Surgery, All India Institute of Medical Sciences, New Delhi, Delhi, India; 2 Department of Cardiothoracic and Vascular Surgery, VMMC and Safdarjung Hospital, New Delhi, Delhi, India; 3 Department of Cardiac Anaesthesia, All India Institute of Medical Sciences, New Delhi, Delhi, India

**Keywords:** pseudoaneurysm, Masson's hemangioma, IPEH

## Abstract

Intravascular papillary endothelial hyperplasia or Masson's tumor is a rare reactive disease of vascular origin characterized by exuberant proliferation of endothelial cells. Its importance lies in its ability to mimic a variety of diseases, both benign and malignant. Here, we present a unique case of Masson's tumor arising from the abdominal supraceliac aorta in a 32-year-old man initially misdiagnosed as leaking aortic pseudoaneurysm.

## Introduction

Initially named as “hemangioendotheliome vegetant intravasculaire” by Masson 1 in 1923, Masson's hemangioma is an exuberant endothelial proliferation that requires differential diagnosis from angiosarcoma. Presently, it is considered to be a reactive vascular proliferation following traumatic vascular stasis. 2

Although there are a few reports of Masson's hemangioma, also termed intravascular papillary endothelial hyperplasia (IPEH), in normal blood vessels and vascular malformations, we present the first reported case of IPEH mimicking as a leaking pseudoaneurysm of the descending thoracic and abdominal aorta.

## Case Presentation

A 32-year-old man with a history of progressive increase in dyspnea saturation for 1 month presented to a peripheral hospital. On examination he was tachypneic (room air –84%) with decreased air entry on the right side and with normal hemodynamics values. Cardiac auscultation revealed normal heart sounds and no cardiac murmur. A chest X-ray was done which showed a massive right-sided pleural effusion, and a right intercostal tube drainage was done at an outside hospital which drained 1 L of blood immediately and 1 L subsequently.

**Fig. 1:**
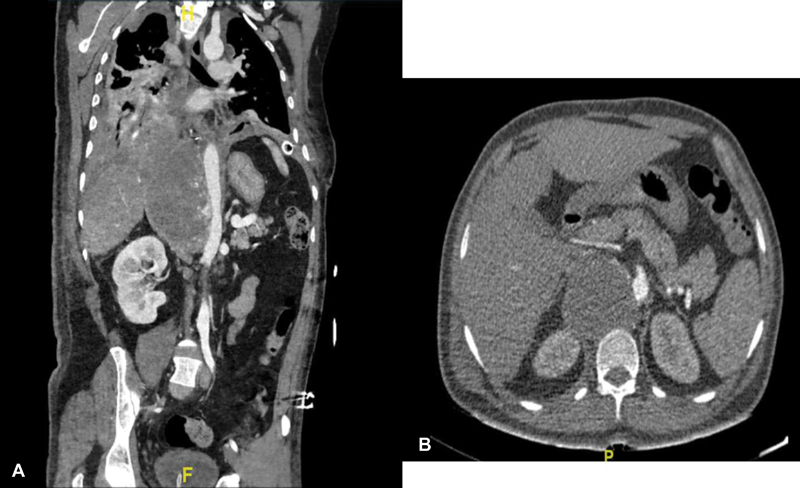
( **A** ) Coronal section computed tomography angiography showing hypodense hematoma extending along the right side of the aorta from the D10 to L1 level with active contrast extravasation (later diagnosed to be collaterals). Also, loculated right pleural effusion is noted. ( **B** ) Axial cut section showing the same lesion receiving collaterals from the adjacent aorta.

The patient was referred to our institute with the diagnosis of massive right hemothorax. An ultrasound of the chest was done which showed gross right pleural effusion with collapse of underlying lung. A well-defined retrocrural collection of size 7 × 6 cm was noted. The liver was normal in echotexture. Emergency computed tomography angiography (CTA) was suggestive of massive right hemothorax, with an aortic rent at the T7 level communicating with the right pleura and multiple aortic rents at the T10–11 level with a surrounding 8 × 4 cm hematoma ( Fig. 1 ).

On detailed history taking, the patient informed us that 6 years ago he had laparoscopic cholecystectomy during which his common bile duct was inadvertently injured and eventually a Roux-en-Y hepaticojejunostomy was done.

With a provisional diagnosis of a leaking pseudoaneurysm, the patient was taken for surgical exploration to repair the aortic rent as well as the communication with right pleura through a left thoracoabdominal approach. After taking proper proximal and distal aortic control, the descending thoracic aorta was opened above the renal arteries, but the aortic wall was found to be normal with no rents. The aorta was closed. Surgical exploration could not be extended on the right side because of dense adhesions due to the past surgery.

The patient was then repositioned and right thoracotomy done, which showed a 2 × 2 cm laceration on right lower lobe of the lung from which blood was draining freely, probably sustained during chest tube insertion. This was repaired. No other source of bleeding could be identified. Postoperatively, drainage from chest tube decreased but did not subside. CTA was repeated, which again revealed the hematoma of the same size with some contrast leak just above the celiac artery on the anterolateral wall of the aorta. Thus, the decision was taken to stent the descending thoracic aorta above the celiac artery level.

Stenting of the descending thoracic aorta was done via the right femoral artery approach under fluoroscopic guidance from just below the arch vessels to just above the celiac axis. However, even poststenting blood drainage did not subside, and serial chest X-rays showed increasing collection in the right pleural cavity with underlying lung collapse.

**Fig. 2:**
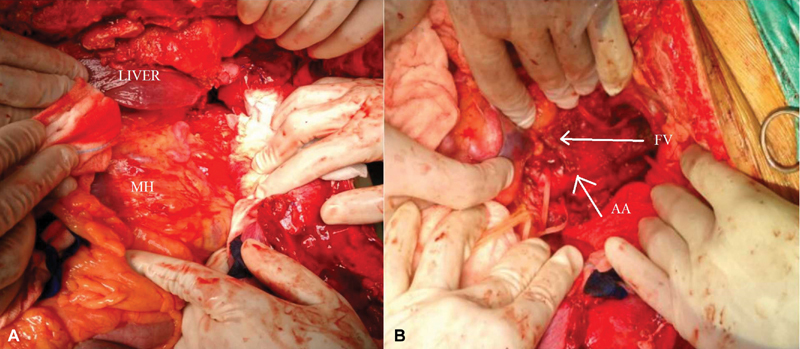
( **A** , **B** ) Intraoperative photograph after right subcostal incision showing tumor mass with feeding vessels from the aorta. AA, abdominal aorta; FV, feeding vessels; MH, Masson's hemangioma.

A decision was taken to re-explore through a right subcostal incision. The liver was mobilized and a mass of size 8 × 3 × 2 cm was identified in the right paravertebral region near the crux of the right diaphragm with bleeding found to be occurring from the mass, which was excised using a combination of electric cautery and blunt dissection ( Fig. 2 ). Multiple feeding vessels to the mass were present arising from the aorta and the aortic wall was completely normal.

**Fig. 3:**
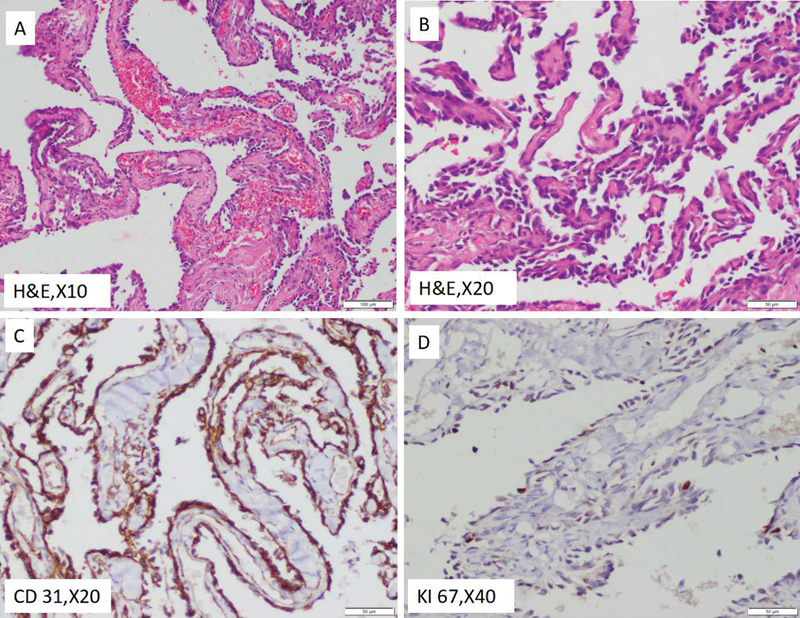
( **A** , **B** ) The lesion is composed of multiple papillae lined by bland endothelial cells. Features are those of papillary endothelial hyperplasia (Masson's hemangioma). ( **C** , **D** ) Immunohistochemically, the lining cells are positive for CD 31 with very low proliferative index (less than 5%).

The mass was sent for histopathological examination. The lesion was composed of multiple papillae lined by bland endothelial cells suggestive of papillary endothelial hyperplasia (Masson's hemangioma) ( Fig. 3 ).

Postoperatively, the drainage settled but the patient required prolonged ventilator support and eventually succumbed to sepsis due to ventilator-associated pneumonia after a prolonged hospital stay of 3 months.

## Discussion

IPEH is an unusual intravascular process that bears remarkable resemblance to hemangiosarcoma. It has various names, including Masson's tumor, Masson's hemangioma, Masson's intravascular hemangioendothelioma, IPEH, and reactive papillary endothelial hyperplasia. 3 IPEH may occur in any location in the body, with a predilection for the deep dermis and subcutis of the head, neck, fingers, and trunk. It has no age tendency and shows a slight female predominance with a male/female ratio of 1:1.2. 4 The pathogenesis of papillary endothelial hyperplasia is still unclear, but the exuberant endothelial cell proliferation might be stimulated by an autocrine loop of endothelial basic fibroblast growth factor secretion. 5

Hashimoto et al 4 described three forms of IPEH. The primary form arises in a dilated vascular space, the secondary or mixed form occurs within a preexisting vascular lesion, and the third or extravascular form appears within the hematomas. Pins et al documented 314 cases, with the primary form being the most common. 6 In the present case, the patient had a history of trauma in the form of surgery in the past, which might have resulted in a hematoma formation eventually leading to Masson's hemangioma; so, this can be considered as a secondary form of IPEH in our patient.

Masson's hemangioma often poses a diagnostic challenge, as the clinical signs and symptoms are nonspecific and may simulate a soft tissue sarcoma. The presence of an exuberant papillary growth in IPEH may closely simulate the tufting growth of angiosarcoma. 3 On analyzing the clinical course of our patient retrospectively, we found that the contrast leak on CTA, which was present despite interventions including surgery and aortic stenting, was due to the feeding vessels of the hemangioma arising directly from the aorta.

IPEH is a nonneoplastic reactive lesion within blood vessels associated with thrombi. As the clinical and radiological signs are nonspecific, IPEH, like other vascular malformations, poses a diagnostic challenge. The purpose of this report is to illustrate the misdiagnosis associated with this condition, as happened in our case of IPEH simulating clinically and radiologically as leaking aortic pseudoaneurysm. Histological examination provides the mainstay in achieving the correct diagnosis. Although the treatment for IPEH is complete surgical excision, achieving a correct diagnosis is essential to avoid subjecting a patient to unnecessary aggressive therapy.
